# Comparing Inpatient Complication Rates between Octogenarians and Nonagenarians Following Primary and Revision Total Hip Arthroplasty in a Nationally Representative Sample 2010–2014

**DOI:** 10.3390/geriatrics4040055

**Published:** 2019-10-01

**Authors:** Evan M Dugdale, David Tybor, Michael Kain, Eric L Smith

**Affiliations:** 1 Boston University School of Medicine, 72 E Concord St, Boston, MA 02118, USA; 2 Tufts University School of Medicine, 145 Harrison Ave, Boston, MA 02111, USA; 3 Department of Orthopedics, Boston Medical Center, One Boston Medical Center Pl, Boston, MA 02118, USA; 4 New England Baptist Hospital, 125 Parker Hill Ave, Boston, MA 02120, USA

**Keywords:** total hip arthroplasty, total hip revision arthroplasty, arthroplasty in octogenarians, arthroplasty in nonagenarians, primary total hip arthroplasty complications, revision total hip arthroplasty complications

## Abstract

We compared inpatient postoperative complication rates between octogenarians and nonagenarians undergoing primary and revision total hip arthroplasty (THA). We used inpatient admission data from 2010–2014 from the Nationwide Inpatient Sample (NIS). We compared the rates at which nonagenarians and octogenarians developed each complication in the inpatient setting following both primary THA (PTHA) and revision THA (RTHA). A total of 40,944 inpatient admissions were included in our study which extrapolates to a national estimate of 199,793 patients. A total of 185,799 (93%) were octogenarians and 13,994 (7%) were nonagenarians. PTHA was performed on 155,669 (78%) and RTHA was performed on 44,124 (22%) of the patients. Nonagenarians undergoing PTHA required transfusions significantly more frequently (33.13% v. 24.0%, *p* < 0.001) and developed urinary tract infection (5.14% v. 3.92%, *p* = 0.012) and acute kidney injury (5.50% v. 3.57%, *p* < 0.001) significantly more frequently than octogenarians. Nonagenarians undergoing RTHA required transfusions significantly more frequently (51.43% v. 41.46%, *p* < 0.001) and developed urinary tract infection (19.66% v. 11.73%, *p* < 0.001), acute kidney injury (13.8% v. 9.66%, *p* < 0.001), pulmonary embolism (1.24% v. 0.67%, *p* = 0.031), postoperative infection (1.89% v. 1.11%, *p* = 0.023), sepsis (3.59% v. 2.43%, *p* = 0.021) and other postoperative shock (1.76% v. 1.06%, *p* = 0.036) significantly more frequently than octogenarians. Nonagenarians undergoing RTHA also had a significantly higher inpatient mortality rate (3.28% v. 1.43%, *p* < 0.001) than octogenarians. Orthopedic surgeons and primary care providers can use these findings to help counsel both their octogenarian and nonagenarian patients preoperatively when considering THA. Our analysis can help these patients better understand expected inpatient complication rates and assist them in deciding whether to pursue surgical intervention when applicable.

## 1. Introduction

The age demographics of the United States are changing, with the elderly continuing to constitute an expanding proportion of the population. By 2060, almost 25% of the United States population is projected to be composed of patients over age 65 and between 2010 and 2050 the number of Americans aged 90 years and older is expected to quadruple [1,2]. The volume of total joint arthroplasties performed on nonagenarians will likely increase rapidly as this age cohort grows in number. However, some orthopedic surgeons may be reluctant to perform total joint arthroplasty on a nonagenarian due to the significant number of medical comorbidities expected in this age demographic [3]. It is therefore important that orthopedic surgeons as well as primary care providers and other members of the care team have an understanding of the expected postoperative inpatient course of nonagenarians undergoing total joint arthroplasty. Orthopedic surgeons, in particular, could use this knowledge to more efficiently direct focused efforts at reducing common postoperative inpatient complications and better guide their conversations with patients preoperatively when discussing whether or not to pursue surgery.

Our group recently published a paper in *Geriatrics* that compared select inpatient complication rates between octogenarians and nonagenarians following total knee arthroplasty (TKA). One arm of our study looked at patients following primary TKA and another arm looked at patients following revision TKA. In both cases, we identified several postoperative complications that occurred at significantly higher rates in the nonagenarian group than the octogenarian group. In the current paper, we used the same study design but compared inpatient complication rates of octogenarians and nonagenarians following total hip arthroplasty (THA) rather than TKA.

Several groups have studied the outcomes of octogenarian and nonagenarian patients undergoing THA. Nanjayan et al. tracked inpatient complications developed by 101 patients aged 80 and older undergoing primary total hip arthroplasty at a single center. Pneumonia, hypotension, confusion/delirium and urinary tract infection were the most commonly developed complications, each occurring in 4% of patients. This study helped to provide an idea of common inpatient complications and complication rates, however the relatively small sample size of this study and the fact that it was performed at a single center limit its generalizability. In addition, this study did not compare outcomes between octogenarians and nonagenarians [4].

Clement et al. performed another single center study that compared the outcomes after primary THA of 163 patients age 80 years and older against 376 patients aged 65–74. Notably, the rates of transfusion, confusion, pneumonia, urinary tract infection and myocardial infarction were all significantly higher in the older cohort as was the length of stay. This study benefitted from a larger sample size than the Nanjayan et al. paper and also provided some insight into the relative risks of an older age cohort compared to a younger age cohort. However, the study design does not permit any conclusions to be made about the outcomes of specifically nonagenarians [5].

Miric et al. performed a retrospective analysis of a large total joint replacement registry and compared the outcomes following primary THA of 183 nonagenarians to those of 4725 octogenarians and 38,620 patients less than eighty years old. This study provided excellent insight into the perioperative and postoperative courses of these different age groups and specifically compared the outcomes of nonagenarians and octogenarians following primary. However, this group looked solely at the outcomes of patients undergoing primary THA and did not look at the outcomes of patients following revision total hip arthroplasty [6].

Building on these prior studies, we used a large nationally representative dataset to compare the postoperative inpatient complication rates of octogenarians and nonagenarians undergoing primary THA and revision THA. By comparing these two adjacent age groups, our results can help illustrate the incremental risks associated with nonagenarians compared to octogenarians following both operations. These data can help patients and providers alike better understand the marginal risks in postoperative inpatient complications that nonagenarians may face as compared to their younger counterparts.

The purpose of this study was to compare inpatient complication rates of octogenarians and nonagenarians following both primary and revision THA.

## 2. Materials and Methods 

We used the Nationwide Inpatient Sample (NIS) to gather data on both primary total hip arthroplasty (PTHA) and revision total hip arthroplasty (RTHA) procedures performed between 2010 and 2014. The NIS uses data collected prospectively from a stratified sample comprising 20% of all US community hospital discharges. National estimates can be extrapolated from the sample using NIS-provided sample weights. Approximately 97% of the United States population is represented through the NIS with 46 states and the District of Columbia contributing to the dataset. We analyzed data from 2010 to 2014 and used NIS-provided guidelines for aggregating the annual samples [7].

We used ICD-9 codes to isolate patients undergoing the procedures of interest, PTHA and RTHA. Patients with the ICD-9 Procedure Code 81.51 (total hip replacement) and a primary diagnosis of osteoarthritis were included in the PTHA group. The ICD-9 Diagnosis Codes used to identify patients with a primary diagnosis of osteoarthritis were 715.15 (localized primary osteoarthritis, pelvic region and thigh), 715.25 (localized secondary osteoarthritis, pelvic region and thigh), 715.35 (primary osteoarthritis, not specified whether primary or secondary, pelvic region and thigh) and 715.95 (osteoarthritis, unspecified whether generalized or localized, pelvic region and thigh). Patients matching 00.70 (revision of hip replacement, both acetabular and femoral components), 00.71 (revision of hip replacement, acetabular component), 00.72 (revision of hip replacement, femoral component), 00.73 (revision of hip replacement, acetabular liner and/or femoral head only), or 81.53 (revision of hip replacement, not otherwise specified) were included in the RTHA group. Any records matching both ICD-9 Procedure codes for primary and revision total hip arthroplasty were excluded from the study.

Using the NIS-provided patient age in years, we generated one treatment group of patients aged 80–89 years and one treatment group of patients aged 90–99 years. 

NIS-provided metrics (e.g., length of stay, total charges, number of chronic conditions, etc.) were used to identify patient characteristics and outcomes. Patient complications sustained in the inpatient setting were identified using ICD-9 diagnosis and procedure codes. Specifically, ICD-9 codes indicating transfusion, deep venous thrombosis (DVT), pulmonary embolism (PE), postoperative infection, acute myocardial infarction (MI), wound dehiscence, pneumonia/pneumonitis, urinary tract infection (UTI), cerebrovascular accident (CVA), acute kidney injury (AKI), cardiogenic shock, other postoperative shock, and sepsis were used to identify these complications of interest (see Appendix A). We also used the Enhanced ICD-9 code method developed by Quan et al. to calculate a Charlson Comorbodity Index value for each inpatient record [8].

To compare the characteristics and outcomes of octogenarians and nonagenarians undergoing PTHA and RTHA we used *t*-tests and the chi-square test. Binary logistic regression was used to compare the odds of a patient developing any complication (transfusion, deep venous thrombosis (DVT), pulmonary embolism (PE), postoperative infection, acute myocardial infarction (MI), wound dehiscence, pneumonia/pneumonitis, urinary tract infection (UTI), cerebrovascular accident (CVA), acute kidney injury (AKI), cardiogenic shock, other postoperative shock, and sepsis), or suffering inpatient mortality between octogenarians and nonagenarians. These odds were also compared after adjusting for the following confounding variables: Charlson Comorbidity Index, gender, race, median household income quartile of patient’s zip code, number of diagnoses, number of chronic diagnoses, number of inpatient procedures, month of admission, year of admission, and whether or not admission was on a weekend. All analyses were conducted using Stata 15 (StataCorp, College Station, TX, USA) with a two-sided alpha level of 0.05. Some variables were noted to have a significant positive skew (Length of Stay, Total Charges, and Number of Procedures). To determine significant differences between groups in these variables, we applied a log transformation, and then compared the means between groups with the log-transformed data. Finally, Stata survey commands (svy) were used to adjust for the sampling design of the NIS dataset [9].

## 3. Results

Between 2010 and 2014, a total of 40,944 hospital admission records were included in our study. These records provide a national estimate of the inpatient admissions of 199,793 patients after accounting for NIS-provided sampling weights. Of these admissions, 185,799 (93%) were octogenarian patients and 13,994 (7%) were nonagenarian patients. PTHA was performed on 155,669 (78%) of the patients and RTHA was performed on 44,124 (22%) of the patients.

### 3.1. Primary THA

Nonagenarians undergoing PTHA had more chronic conditions (5.63 vs. 5.45, *p* = 0.007), total number of diagnoses (9.42 vs. 8.94, *p* ≤ 0.001) and number of procedures than octogenarians (2.06 vs. 1.97, *p* ≤ 0.001) (Table 1). A higher percentage of nonagenarians were female compared to octogenarians (70.27% vs. 66.26%, *p* ≤ 0.001). Nonagenarians and octogenarians did not differ significantly in their racial composition or their distributions of median household income or Charlson Comorbidity Index strata.

### 3.2. Primary THA

Nonagenarians undergoing PTHA experienced a significantly longer length of stay (3.83 days vs. 3.45 days, *p* ≤ 0.001) and had higher total charges ($57,704 vs. $55,558, *p* = 0.007) than octogenarians (Table 2). A significantly higher proportion of nonagenarians received transfusions (33.13% vs. 24.0%, *p* ≤ 0.001), experienced a UTI (5.14% vs. 3.92%, *p* = 0.012) and developed AKI (5.50% vs. 3.57%, *p* ≤ 0.001) during their admission compared to octogenarians. The two groups did not differ significantly in the rates at which they developed inpatient mortality or developed deep venous thrombosis, pulmonary embolism, postoperative infection, acute myocardial infarction, wound dehiscence, pneumonia or pneumonitis, cerebrovascular accident, cardiogenic shock, other shock, or sepsis.

We performed a binary logistic regression to compare the odds of a patient developing any of the inpatient complications we evaluated between the octogenarian and nonagenarian cohorts undergoing PTHA. Nonagenarians had significantly higher odds of developing any inpatient complication compared to octogenarians (OR = 1.56, 95% CI: 1.41, 1.73). This finding was unchanged (OR = 1.50, 95% CI: 1.32, 1.71) after accounting for likely confounding variables, including Charlson Comorbidity Index, gender, race, median household income quartile of patient’s zip code, number of diagnoses, number of chronic diagnoses, number of inpatient procedures, month of admission, year of admission, and whether or not admission was on a weekend.

In addition, we performed a binary logistic regression to compare the odds of a patient suffering inpatient mortality between octogenarians and nonagenarians undergoing PTHA. There was no significant difference (OR = 1.02, 95% CI: 0.32, 3.26) in the odds of either group suffering inpatient mortality. This finding was unchanged (OR = 0.86, 95% CI: 0.20, 3.69) after accounting for likely confounding variables, including Charlson Comorbidity Index, gender, race, median household income quartile of patient’s zip code, number of diagnoses, number of chronic diagnoses, number of inpatient procedures, month of admission, year of admission, and whether or not admission was on a weekend.

### 3.3. Revision THA

Nonagenarians undergoing RTHA had more chronic conditions (6.22 vs. 6.00, *p* = 0.016), number of diagnoses (13.05 vs. 12.08, *p* ≤ 0.001) and number of procedures (2.96 vs. 2.80, *p* ≤ 0.001) than octogenarians (Table 3). A higher percentage of nonagenarians were female compared to octogenarians (73.29% vs. 65.68%, *p* ≤ 0.001). The two groups did not differ significantly in their racial compositions or their distributions into median household income or Charlson Comorbidy Index strata.

Nonagenarians undergoing RTHA experienced a significantly longer length of stay (6.78 days vs. 5.70 days, *p* = <0.001) and had higher total charges ($93,170 vs. $83,132, *p* = <0.001) than octogenarians (Table 4). Nonagenarians also developed inpatient mortality (3.28% vs. 1.43%, *p* = <0.001) at a significantly higher rate. Nonagenarians required transfusions (51.43% vs. 41.46%, *p* = <0.001) and developed pulmonary embolism (1.24% vs. 0.67%, *p* = 0.031), postoperative infection (1.89% vs. 1.11%, *p* = 0.023), urinary tract infection (19.66% vs. 11.73%, *p* = <0.001), acute kidney injury (13.8% vs. 9.66%, *p* = <0.001), other postoperative shock (1.76% vs. 1.06%, *p* = 0.036) and sepsis (3.59% vs. 2.43%, *p* = 0.021) at significantly higher rates than octogenarians.

We performed a binary logistic regression to compare the odds of a patient developing any of the inpatient complications we evaluated between the octogenarian and nonagenarian cohorts undergoing RTHA. Nonagenarians had significantly higher odds of developing any inpatient complication compared to octogenarians (OR = 1.77, 95% CI: 1.55, 2.01). This finding was unchanged (OR = 1.73, 95% CI: 1.47, 2.03) after accounting for likely confounding variables, including Charlson Comorbidity Index, gender, race, median household income quartile of patient’s zip code, number of diagnoses, number of chronic diagnoses, number of inpatient procedures, month of admission, year of admission, and whether or not admission was on a weekend.

In addition, we performed a binary logistic regression to compare the odds of a patient suffering inpatient mortality between octogenarians and nonagenarians undergoing RTHA. Nonagenarians were significantly more likely (OR = 2.34, 95% CI: 1.61, 3.40) to suffer inpatient mortality compared to octogenarians. This finding was unchanged (OR = 2.38, 95% CI: 1.54, 3.66) after accounting for likely confounding variables including Charlson Comorbidity Index, gender, race, median household income quartile of patient’s zip code, number of diagnoses, number of chronic diagnoses, number of inpatient procedures, month of admission, year of admission, and whether or not admission was on a weekend.

## 4. Discussion

As nonagenarians continue to make up an increasing proportion of the United States population, they will likely undergo an increasing number of total hip arthroplasties. In this study, we used a large nationally representative dataset to compare the inpatient complication rates of nonagenarians and octogenarians following both primary and revision total hip arthroplasty. This study serves as a complement to our previously published study comparing the inpatient complication rates of octogenarians and nonagenarians following total knee arthroplasty [10].

We found no significant difference in inpatient mortality rate between octogenarians and nonagenarians undergoing PTHA (0.18% vs. 0.19%), however, nonagenarians undergoing RTHA had a significantly higher inpatient mortality rate than octogenarians (3.28% vs. 1.43%), even after controlling for likely confounding variables including number of diagnoses, number of chronic diagnoses and Charlson Comorbidity Index.

These findings can be compared to previously published assessments of inpatient mortality following THA. Using NIS data from 1988–2000, Doro et al. compared hospitals with different levels of THA volume on inpatient mortality rates of patients of all ages following PTHA and RTHA. Inpatient mortality rates ranged from 0.16% to 0.29% following PTHA depending on the hospital’s THA volume. Similarly, inpatient mortality rates following RTHA ranged between 0.48% and 1.2%. Our own calculated inpatient mortality rates, particularly for RTHA, are likely higher due to the higher average age of our patients as compared to the patients in their study. In fact, in the same paper, this group reported that the odds of developing inpatient mortality were 6.9 and 7.6 times higher in the oldest age cohort compared to the youngest age cohort undergoing PTHA and RTHA respectively [11].

Nonagenarians were significantly more likely to require a transfusion and to develop urinary tract infection and acute kidney injury following both PTHA and RTHA. These findings aligned with those previously described by Jauregui et al. They reported that nonagenarians undergoing total joint arthroplasty were at higher risk of developing urinary tract infection and requiring transfusion when compared to a younger cohort [12]. Following RTHA, nonagenarians were also more likely to develop pulmonary embolism, postoperative infection, sepsis and other postoperative shock. It should be noted however that these additional differences in complication rates seen between the two age groups following RTHA were associated with relatively high *p* values compared to the *p* values < 0.001 observed with many of the other significant differences we found between the two age cohorts.

Nonagenarians had a longer average length of stay than octogenarians following both PTHA (3.83 days vs. 3.45 days) and RTHA (6.78 days vs. 5.70 days). These calculations align with several previously described. Miric et al. also found nonagenarians undergoing PTHA to have a longer length of stay than octogenarians (3.4 days vs. 3.3 days) [6]. Bozic et al. and Gwam et al. calculated the average lengths of stay of patients following RTHA to be 6.2 and 5.29 days respectively [13,14].

Nonagenarians also had significantly higher total charges compared to octogenarians following both PTHA and RTHA. D’Apuzzo et al. similarly found that patients 90 years and older had higher total charges than younger patients following primary total joint arthroplasty [15]. The higher total charges and longer length of stay seen in nonagenarians is likely attributable to the higher risk of developing complications in nonagenarians compared to octogenarians and the increased amount of time and money subsequently needed to manage these complications.

As bundled payment models continue to gain traction, a better understanding of the factors that predict the development of complications (and therefore additional costs) will be necessary to ensure the economic sustainability of the model. Our regression analysis showed that nonagenarians had significantly higher odds of developing a complication following both PTHA and RTHA compared to octogenarians. Interestingly, although the nonagenarians did have more diagnoses and chronic conditions than octogenarians undergoing both PTHA and RTHA, this finding was unchanged after controlling for underlying comorbidities through the Charlson Comorbidity Index. This suggests that age may be an important risk factor, independent of underlying comorbidity, to include when attempting to gauge a patient’s risk of developing postoperative complications and thereby incurring higher total charges during their episode of care.

In particular, we reason that increased age led to higher total charges due to the relative susceptibility of our older cohort compared to our younger cohort. As we discussed in our similarly constructed study on total knee arthroplasty, there is likely a substantial difference in the short-term mortality rate between our octogenarian and nonagenarian cohorts. This is best demonstrated by examining the “Death Probability” statistics published by the Social Security Administration (SSA). The SSA’s Death Probability calculates the percentage chance of someone of a certain age dying within the next year. Their data for the year 2014 shows that while an 80-year-old man has a 5.9% chance of dying within the next year, a 90-year-old man has a 16.5% chance. Similarly, while an 80-year-old woman has a 4.3% chance of dying in the next year, a 90-year-old woman has a 12.9% chance. These statistics show how the older cohort in our study is likely more susceptible to short-term mortality at baseline and thus more susceptible to developing complications requiring more expensive management [9].

It is therefore likely that even relatively minor complications, such as the development of a urinary tract infection or the need for a transfusion, could have manifested more severely in our older cohort than our younger cohort. In the nonagenarian cohort, recovery from these complications could have taken longer and required more resources than in the octogenarian cohort since the nonagenarian cohort has overall such a higher baseline likelihood of short-term mortality, as evidenced by the Death Probability discussed above. This on top of the fact that the nonagenarians developed select complications at a significantly higher rate would explain the differences in total charges by a combination of both the quantity of complications developed by nonagenarians and their severity.

Comparing our findings to our previous study on inpatient complications in octogenarians and nonagenarians following total knee arthroplasty, there are several notable similarities and differences. First, nonagenarians had a significantly higher rate of developing urinary tract infection and acute kidney injury and requiring a transfusion following both primary and revision total knee arthroplasty, which is the same pattern we observed following both primary and revision total hip arthroplasty. Similarly, nonagenarians also had a longer length of stay and higher total charges following both primary and revision total knee arthroplasty, as we observed following both primary and revision total hip arthroplasty. However, nonagenarians had significantly higher inpatient mortality following both primary and revision total knee arthroplasty, whereas in our current evaluation of total hip arthroplasty, nonagenarians only had a significantly higher inpatient mortality rate following RTHA. This suggests that the marginal risk of nonagenarians compared to octogenarians of developing inpatient mortality is relatively less dramatic following primary total hip arthroplasty compared to primary total knee arthroplasty [10].

It was not unexpected that our two studies showed similar patterns in the specific complications that occurred at a significantly higher rate in nonagenarians compared to octogenarians. The two procedures, THA and TKA are similar enough in terms of their impact on the patient to produce these similar results. As was discussed prior, it follows that we would expect similar patterns in our two studies when comparing length of stay and total charges. However, it was surprising that while nonagenarians had significantly higher inpatient mortality rates than octogenarians following both primary and revision total knee arthroplasty, the same pattern did not hold in our current THA study. Rather, nonagenarians demonstrated a significantly higher inpatient mortality rate following solely RTHA, with no difference demonstrated following PTHA.

We cannot say definitively what drove this difference between our two studies. The finding suggests that nonagenarians are relatively better equipped to recover in the inpatient setting from a primary total hip arthroplasty than a primary total knee arthroplasty compared to a slightly younger cohort. This could be attributable to a host of perioperative factors inherent to the two procedures and could also include differences in the patients undergoing each procedure. There may be preoperative characteristics of the nonagenarians undergoing PTHA that renders them less susceptible to inpatient mortality than the nonagenarians undergoing primary total knee arthroplasty. Further research could focus on comparing these two groups for differences in their preoperative comorbidities and baseline level of health to determine if such a discrepancy exists and could better explain this difference between our two studies.

The major limitation of our study is that its validity is contingent on the accuracy of the patient’s admission data. Furthermore, our ability to accurately capture the records and complications of interest relies on our use of appropriate ICD-9 Diagnosis and Procedure codes. To capture the records of interest, we used codes previously described with the exception that we did not include ICD-9 Procedure code 80.05 (arthrotomy/removal of prosthesis) as one of our codes to detect a RTHA [11,13]. We felt this decision would improve our specificity in detecting RTHA records, although we acknowledge it may have reduced our sensitivity. To identify the complications of interest, we predominantly used our own investigation to determine the most applicable ICD-9 diagnosis codes. In the case of sepsis, however, we used the ICD-9 codes previously described by Vogel et al. [16]. It is possible that there are other combinations of ICD-9 codes that would have more accurately captured the procedures and diagnoses of interest. In addition, the NIS does not permit us to measure the severity of each complication developed by each patient. As discussed above, some of the differences in length of stay and total charges between our two cohorts may be attributable to increased severity of complications developed by the relatively more susceptible nonagenarians, however, we cannot confirm that through the NIS dataset.

An additional limitation of our study is the fact that we are at risk of committing Type I errors given our large sample size. Miniscule differences can be interpreted as statistically significant at the 5% alpha level given our significantly large sample size. With this in mind, it is important to evaluate our findings for true clinical relevance. For instance, although nonagenarians undergoing RTHA developed pulmonary embolism at a rate significantly higher than octogenarians, the real difference in complication rate is fairly small (1.24% vs. 0.67%). Further evaluation would be necessary to determine if this statistically significant difference is of any clinical relevance.

Our study’s major strengths stem from our use of a large nationally representative dataset. With an n value close to 200,000, our findings have high precision. Furthermore, since the NIS dataset is nationally representative, our study has a wide generalizability.

## 5. Conclusions

In conclusion, our study suggests that nonagenarian patients had longer lengths of stay, higher total charges and were more likely to develop select complications than octogenarians following both PTHA and RTHA. Many of the complications developed were minor, with transfusion, urinary tract infection and acute kidney injury being the most common. Although nonagenarians developed several more major complications at significantly higher rates than octogenarians following RTHA, further investigation is necessary to determine the clinical relevance of these additional discrepancies given the relatively high *p* values associated with these findings in the setting of the exceptionally large sample size used in our study. Moving forward, our study can help orthopedic surgeons and primary care providers in counseling both their octogenarian and nonagenarian patients on the postoperative inpatient risks of total hip arthroplasty and assist patients in their decision-making process when considering the pursuit of surgical intervention.

## Figures and Tables

**Table 1:** Comparing characteristics of patients undergoing primary total hip arthroplasty between octogenarians and nonagenarians. Healthcare Cost and Utilization Project—Nationwide Inpatient Sample, 2010–2014.

	Octogenarians (n = 147,281)	Nonagenarians (n = 8388)	*p* Value
Age (years), mean (SD)	83.19 (2.53)	90.55 (1.27)	-
Number of Chronic Conditions, mean (SD)	5.45 (2.55)	5.63 (2.59)	0.007
Number of Diagnoses, mean (SD)	8.94 (4.50)	9.42 (4.61)	<0.001
Number of Procedures, mean (SD)	1.97 (1.10)	2.06 (1.03)	<0.001 *
% Female	66.26	70.27	<0.001
Race			0.198
% White	91.77	92.56	
% Black	3.11	2.18	
% Hispanic	2.46	2.15	
% Asian or Pacific Islander	0.81	1.16	
% Native American	0.24	0.33	
% Other	1.61	1.62	
Median Household Income (1=lowest quartile, 4 = highest quartile)			0.095
% 1	18.58	16.98	
% 2	26.37	25.98	
% 3	27.26	26.63	
% 4	27.79	30.41	
Charlson Comorbidity Index			0.055
% 4	53.63	53.65	
% 5	24.89	22.85	
% 6+	21.48	23.51	

* *t*-test performed on log-transformed data to meet the assumption of normality.

**Table 2:** Comparing the outcomes and complications of patients undergoing primary total hip arthroplasty between octogenarians and nonagenarians. Healthcare Cost and Utilization Project—Nationwide Inpatient Sample, 2010–2014.

	Octogenarians (n = 147,281)	Nonagenarians (n = 8388)	*p*-Value
Length of Stay (days), mean (SD)	3.45 (1.89)	3.83 (2.41)	<0.001 *
Total Charges (USD), mean (SD)	55,558 (31,717)	57,704 (31,213)	0.007 *
% Hospital Mortality	0.18	0.19	0.977
% Receiving Transfusion	24.0	33.13	<0.001
% DVT	0.11	0.12	0.895
% PE	0.24	0.22	0.861
% Postop Infection	0.04	0.0	0.390
% Acute MI	0.61	0.94	0.067
% Dehiscence	0.02	0.0	0.528
% Pneumonia or Pneumonitis	0.44	0.70	0.116
% UTI	3.92	5.14	0.012
% CVA	0.10	0.18	0.371
% AKI	3.57	5.50	<0.001
% Cardiogenic Shock	0.02	0.06	0.202
% Other Shock	0.16	0.35	0.059
% Sepsis	0.24	0.18	0.641

* *t*-test performed on log-transformed data to meet the assumption of normality.

**Table 3:** Comparing characteristics of patients undergoing revision total hip arthroplasty between octogenarians and nonagenarians. Healthcare Cost and Utilization Project—Nationwide Inpatient Sample, 2010–2014.

	Octogenarians (n = 38,518)	Nonagenarians (n = 5606)	*p* Value
Age (years), mean (SD)	83.70 (2.65)	90.70 (1.59)	-
Number of Chronic Conditions, mean (SD)	6.00 (2.90)	6.22 (2.87)	0.016
Number of Diagnoses, mean (SD)	12.08 (5.41)	13.05 (5.37)	< 0.001
Number of Procedures, mean (SD)	2.80 (1.82)	2.96 (1.74)	< 0.001 *
% Female	65.68	73.29	< 0.001
Race			0.334
% White	91.89	92.23	
% Black	2.97	2.12	
% Hispanic	2.61	2.63	
% Asian or Pacific Islander	0.59	1.05	
% Native American	0.45	0.39	
% Other	1.49	1.59	
Median Household Income (1 = lowest quartile, 4 = highest quartile)			0.428
% 1	20.17	19.97	
% 2	25.84	26.65	
% 3	27.60	25.47	
% 4	26.39	27.91	
Charlson Comorbidity Index			0.202
% 4	45.40	44.05	
% 5	25.43	24.19	
% 6+	29.16	31.76	

* *t*-test performed on log-transformed data to meet the assumption of normality.

**Table 4:** Comparing the outcomes and complications of patients undergoing revision total hip arthroplasty between octogenarians and nonagenarians. Healthcare Cost and Utilization Project—Nationwide Inpatient Sample, 2010–2014.

	Octogenarians (n = 38,518)	Nonagenarians (n = 5606)	*p*-Value
Length of Stay (days), mean (SD)	5.70 (4.67)	6.78 (4.55)	<0.001 *
Total Charges (USD), mean (SD)	83,132 (62,223)	93,170 (67,890)	<0.001 *
% Hospital Mortality	1.43	3.28	<0.001
% Receiving Transfusion	41.46	51.43	<0.001
% DVT	0.54	0.98	0.064
% PE	0.67	1.24	0.031
% Postop Infection	1.11	1.89	0.023
% Acute MI	1.24	1.76	0.174
% Dehiscence	0.80	1.23	0.155
% Pneumonia or Pneumonitis	0.56	0.52	0.866
% UTI	11.73	19.66	<0.001
% CVA	0.09	0.08	0.924
% AKI	9.66	13.8	<0.001
% Cardiogenic Shock	0.04	0.0	0.509
% Other Shock	1.06	1.76	0.036
% Sepsis	2.43	3.59	0.021

* *t*-test performed on log-transformed data to meet the assumption of normality.

## Appendix A. ICD-9 Codes Used to Identify Postoperative Complications.

**Complication**	**ICD-9 Code**	**ICD-9 Code Description**
**Transfusion**	99.03	Other transfusion of whole blood
	99.04	Transfusion of packed cells
**Acute Deep Venous Thrombosis of Lower Extremity**	453.40	Acute venous embolism and thrombosis of unspecified deep vessels of lower extremity
	453.41	Acute venous embolism and thrombosis of deep vessels of proximal lower extremity
	453.42	Acute venous embolism and thrombosis of deep vessels of distal lower extremity
**Pulmonary Embolism**	415.1	Pulmonary embolism and infarction
	415.11	Iatrogenic pulmonary embolus and infarction
	415.13	Saddle embolus of pulmonary artery
	415.19	Other pulmonary embolism and infarction
**Postoperative Infection**	998.59	Other postoperative Infection
	998.51	Infected postoperative seroma
	998.5	Postoperative infection not elsewhere classifed
**Acute Myocardial Infarction**	410.01	Acute myocardial infarction of anterolateral wall, initial episode of care
	410.11	Acute myocardial infarction of other anterior wall, initial episode of care
	410.21	Acute myocardial infarction of inferolateral wall, initial episode of care
	410.31	Acute myocardial infarction of inferoposterior wall, initial episode of care
	410.41	Acute myocardial infarction of other inferior wall, initial episode of care
	410.51	True posterior wall infarction, initial episode of care
	410.61	Acute myocardial infarction of anterolateral wall, initial episode of care
	410.71	Subendocardial infarction, initial episode of care
	410.81	Acute myocardial infarction of other specified sites, initial episode of care
	410.91	Acute myocardial infarction of unspecified site, initial episode of care
**Dehiscence**	998.30	Disruption of wound, unspecified
	998.31	Dehiscence of internal operation wound
	998.32	Dehiscence of external operation wound
**Pneumonia or Pneumonitis**	997.39	Postoperative pneumonia or postoperative pneumonitis
	997.32	Postoperative pneumonia or postoperative pneumonitis, aspiration type
**Urinary Tract Infection**	599.0	Urinary tract infection, site not specified
**Cerebrovascular Accident**	997.02	Postoperative cerebrovascular accident
**Acute Kidney Failure**	584	Acute kidney failure
	584.5	Acute kidney failure with lesion of tubular necrosis
	584.6	Acute kidney failure with lesion of renal cortical necrosis
	584.7	Acute kidney failure with lesion of renal medullary (papillary) necrosis
	584.8	Acute kidney failure with other specified pathological lesion of kidney
	584.9	Acute kidney failure, unspecified
**Cardiogenic Shock**	998.01	Postoperative cardiogenic shock
**Other Postoperative Shock**	998.0	Postoperative shock not elsewhere specified
	998.00	Postoperative shock, unspecified
	998.09	Postoperative shock, other
**Sepsis**	998.02	Postoperative septic shock
	038.0	Streptococcal septicemia
	038.10	Staphylococcal septicemia unspecified
	038.11	Staphylococcus aureus septicemia
	038.19	Other staphylococcal septicemia
	038.2	Pneumococcal septicemia
	038.3	Septicemia due to anaerobes
	038.40	Septicemia due to gram-negative organism unspecified
	038.41	Septicemia due to Hemophilus influenza
	038.42	Septicemia due to Escherichia coli
	038.43	Septicemia due to Pseudomonas
	038.44	Septicemia due to Serratia
	038.49	Septicemia due to other gram-negative organisms
	038.8	Other unspecified septicemias
	038.9	Unspecified septicemia
	785.52	Septic shock
	785.59	Other shock without mention of trauma
	995.91	Systemic inflammatory response syndrome due to infectious process without organ dysfunction
	995.92	Systemic inflammatory response syndrome due to infectious process with organ dysfunction
